# Early diagnosis of symptomatic ovarian cancer in primary care in the UK: opportunities and challenges

**DOI:** 10.1017/S146342362200041X

**Published:** 2022-09-02

**Authors:** Sanketh Rampes, Anita Bolina, Shern-Ping Choy

**Affiliations:** 1 Faculty of Life Sciences and Medicine, King’s College London, Guy’s Campus, London SE1 1UL, UK; 2 Department of Medicine, Imperial College London, South Kensington Campus, London SW7 2AZ, UK; 3 Cambridge Academy of Therapeutic Sciences, University of Cambridge, 17 Mill Lane, Cambridge CB2 1RX, UK

**Keywords:** community health services, early diagnosis, ovarian neoplasms, primary health care, UK, women’s health

## Abstract

**Background:**

Ovarian cancer is the sixth most common cause of cancer-related death in the UK amongst women. Ovarian cancer presents particular challenges for general practitioners (GPs) to diagnose due to its rarity and presentation with non-specific symptoms.

**Methods:**

A narrative overview of the literature was conducted by searching PubMed and Researchgate for relevant articles, using keywords such as “ovarian cancer,” “primary care” and “diagnosis.”

**Results and Discussion:**

Studies have shown that in the UK, GPs have a lower readiness to refer and investigate potential cancer symptoms compared with their international counterparts; and this has been correlated with reduced survival. Early diagnosis can be facilitated through a people-focussed and system-based approach which involves both educating GPs and using risk algorithms, rapid diagnostic centres/multi-disciplinary centres and being data-driven through the identification of best practice from national audits. Further research is required into the best evidence-based early investigations for ovarian cancer and more effective biomarkers.

## Introduction

Ovarian cancer is the sixth most common cause of cancer-related death in the UK amongst women, with around 4,200 deaths per year (Cancer Research UK, 2018). The majority of patients with cancer present with symptoms, and the majority of these presentations are in primary care (Hamilton, 2010). Studies have shown a relationship between the early referral of suspected cancer patients by general practitioners (GPs) and cancer survival rates (Round *et al.*, 2020). Ovarian cancer survival rates and cancer survival rates more broadly vary significantly on an international level. The International Cancer Benchmarking Partnership (ICBP) was set up to identify causes of international variation in cancer survival. Factors contributing towards variation in ovarian cancer detection in primary care include: differences in care pathways and access to investigations and tests. A key finding from the ICBP is that on average, UK GPs have a lower readiness to investigate or refer potential cancer symptoms compared to other countries. The ICBP data showed a correlation between lower readiness to refer with lower survival rates, including for ovarian cancer (Rose *et al.*, 2015). The observed association between referral pattern and cancer outcomes highlights an opportunity: targeting primary care referral behaviours could meaningfully change ovarian cancer outcomes.

There are several challenges to the early diagnosis of ovarian cancer: its rarity, non-specific presentation and a lack of high-quality evidence underpinning its early investigation in primary care. Opportunities to increase early diagnosis include: the identification of best practice from the Ovarian Cancer Audit Feasibility Pilot, development of evidence-based interventions in primary care, use of risk algorithms and implementation of rapid diagnostic clinics (RDCs) and multi-disciplinary centres (MDCs). This review outlines the investigation and referral for suspected ovarian cancer, highlights where diagnostic delays can be avoided and explores the challenges and opportunities in the diagnosis of ovarian cancer in primary care.

### Screening

Screening is the process of identifying healthy, asymptomatic individuals who may have an increased chance of disease, with the aim of enabling earlier treatment. Population screening programmes offer significant benefits and harms, which must be carefully evaluated to ensure net benefit measured in lives saved (World Health Organisation, 2020). In the UK, national screening programmes exist for breast, cervical and bowel cancer. For ovarian cancer, the largest randomised controlled study (RCT) of ovarian cancer screening to date, the UK Collaborative Trial of Ovarian Cancer Screening (UKTOCS) suggests that although the rate of early cancer detection was higher in the screening group compared to the no screening group, this approach did not lead to a reduction in ovarian cancer mortality (Menon *et al.*, 2021). Until more effective screening measures are available, population screening cannot be justified. Therefore, this review focusses on the detection of symptomatic ovarian cancer.

### Investigation and referral for suspected ovarian cancer

**Figure 1.**
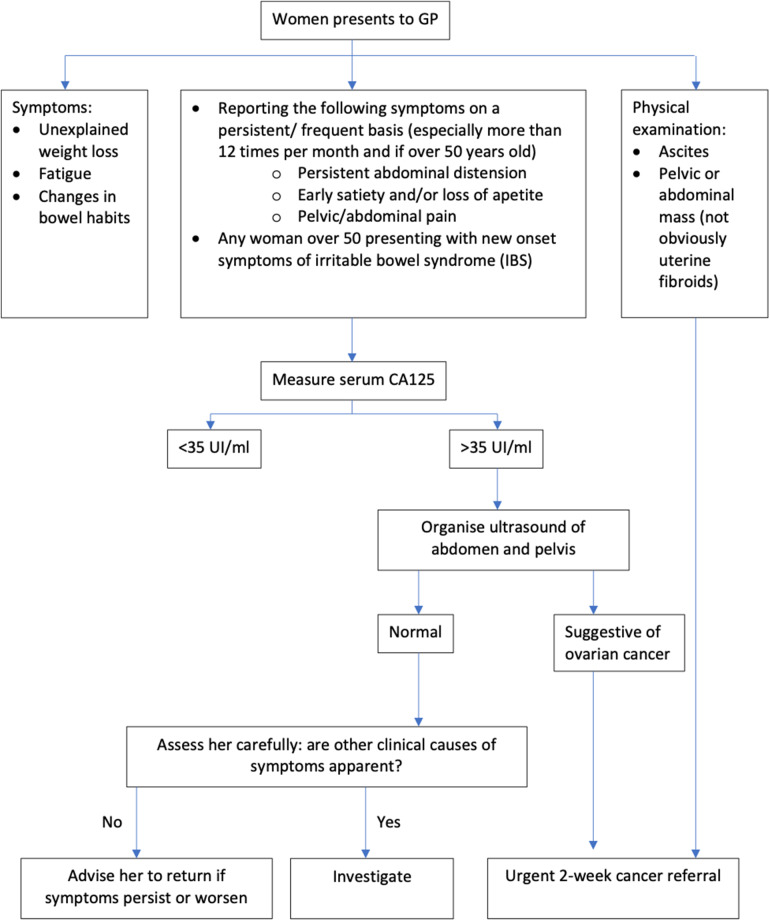
Ovarian cancer: recognition and initial management. Adapted from NICE Clinical guideline [CG122].

The National Institute for Health and Care Excellence (NICE) is an agency of the NHS which was established to provide evidence-based recommendations for healthcare in England. NICE guidelines for referral of suspected ovarian cancer divides pathways depending on the patient’s presenting symptoms (Figure 1). Patients who have specific ovarian cancer signs, such as ascites or a mass in their pelvis or abdomen, should be referred urgently to a specialist, via a 2-week wait pathway. However, most ovarian cancer patients present with non-specific symptoms. For patients who have non-specific but concerning symptoms (NSCS) such as bloating, abdominal pain, weight loss or increased urinary frequency, GPs are recommended to carry out primary care testing. The first test is a measure of serum CA-125. If abnormal, an ultrasound scan of the abdomen and pelvis is recommended. Subsequently, if the ultrasound suggests ovarian cancer, patients will be referred urgently, to be seen by a specialist within 2 weeks (National Institute for Health and Care Excellence, 2011).

### Diagnostic delays for ovarian cancer

**Figure 2.**
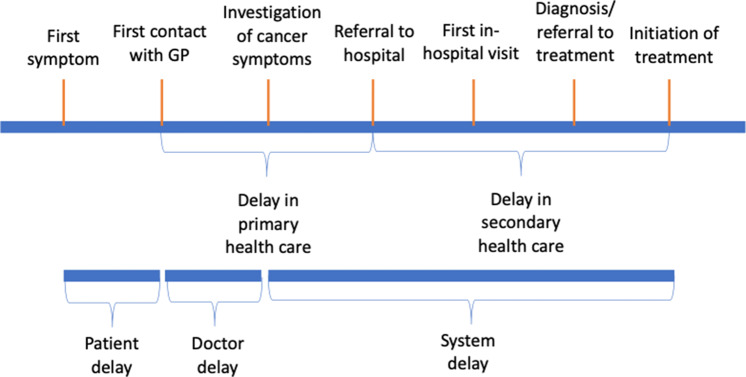
Model of diagnostic delay by Olesen et al. (2009), illustrating the different stages of delay during the process of being diagnosed with cancer.

Diagnostic delays for the cancer care pathway consist of three distinct stages: (1) length of time from symptom onset to first consultation with GP (patient interval); (2) length of time from first consultation to specialist referral (primary care interval); and (3) length of time from secondary care referral to diagnosis (secondary care interval) (Figure 2). For patients with ovarian cancer, the National Cancer Diagnosis Audit (NCDA) dataset reveals that these patients have a median (IQR) patient interval of 14 (2–52) days and a median primary care interval of 7 (0–22) days (Lyratzopoulos *et al.*, 2015). The data are positively skewed with 10% of patients having a primary care interval greater than 51 days (Lyratzopoulos *et al.*, 2015). 36% of patients with ovarian cancer have greater than three primary care consultations before referral to a specialist, which compares poorly to other cancers (Mendonca, Abel and Lyratzopoulos, 2016).

### Challenges

#### Non-specific symptoms/presentation of ovarian cancer

A considerable difficulty in prompt recognition and early referral to specialists is that almost all symptoms associated with ovarian cancer (abdominal pain, bloating, loss of appetite, urinary frequency, post-menopausal bleeding, rectal bleeding) have a positive predictive value of less than 1%, with the exception of abdominal distension at 2.4% (Hamilton *et al.*, 2009) (Table 1). The five-year survival of stage I ovarian cancer is 93% compared to 13% for stage IV (2018). Ovarian cancer is rare, with around 6500 new cases in England each year (2018). The average GP will see a new diagnosis of ovarian cancer once every five years (National Collaborating Centre for Cancer (UK), 2011). Therefore, unlike more common cancers such as breast and lung cancer, GPs build up limited experience in diagnosing ovarian cancer. It is thus prudent that GPs have a lower threshold for suspecting and examining for ovarian cancer. Yet, this must be finely balanced with the negative consequences of overtesting for ovarian cancer, particularly in younger women who are at low risk.

**Table 1.** Odds ratios (95% confidence intervals) and positive predictive values (95% confidence intervals) for the seven symptoms associated with ovarian cancer in multivariate analysis by Hamilton et al. (2009)

Symptom	Odds ratio (95% CI)	*P* value	Positive predictive value (95% CI)
Abdominal distension	240 (46, 1200)	<0.001	2.5 (1.2, 5.9)
Postmenopausal bleeding	24 (9.3, 64)	<0.001	0.5 (0.2, 0.9)
Loss of appetite	17 (6.1, 50)	<0.001	0.6 (0.3, 1.0)
Urinary frequency	16 (5.6, 48)	<0.001	0.2 (0.1, 0.3)
Abdominal pain	12 (6.1, 22)	<0.001	0.3 (0.2, 0.3)
Rectal bleeding	7.6 (2.5, 23)	<0.001	0.2 (0.l, 0.4)
Abdominal bloating	5.3 (1.8, 16)	0.003	0.3 (0.2, 0.6)

#### Balancing people-focussed and systems-focussed behavioural interventions

Another challenge concerning the early diagnosis of ovarian cancer is that UK GPs have a lower readiness to refer patients on to specialists, compared to other countries (Rose *et al.*, 2015). Behavioural interventions to improve ovarian cancer referral rates may facilitate GP behavioural change. However, it can be challenging to design interventions which are effective and can have a long-lasting impact. Therefore, we propose taking an evidence-based hierarchy of interventions approach, which includes both people-focussed and systems-focussed methods (Cafazzo and St-Cyr, 2012).

People-focussed interventions such as education and training will improve early diagnosis because when GPs are aware of when to suspect ovarian cancer, they are likely to refer cases for testing more efficiently. To support GPs in this regard, toolkits exist for both the Royal College of General Practitioners (RCGP) and BMJ Learning. Education empowers individuals and is a motivator for change. However, education interventions often have a short-lived effect and there is a lack of evidence that education in isolation translates into behaviour change (Shuval *et al.*, 2007). The effects of people-focussed interventions are often diluted over time and with rapid staff turnover.

Systems-focussed approaches are more effective in part because they allow for standardisation across GP services. Automation reduces reliance on human behaviour, as well as the need for regular enforcement (Cafazzo and St-Cyr, 2012). Systems-focussed approaches include risk algorithms that are integrated seamlessly into GP computer systems, as well as MDCs which incorporate automation, computerisation and standard protocols (Cafazzo and St-Cyr, 2012).

#### Lack of research comparing initial investigations for ovarian cancer

Another challenge in early ovarian cancer diagnosis is the lack of comparable evidence on the effectiveness of the two main initial investigations: CA-125 versus a transvaginal ultrasound scan (TVUS). A recent systematic review highlighted this by comparing international guidelines on the investigation of ovarian cancer and found significant variation between countries (Funston *et al.*, 2019). There is a lack of direct evidence comparing the effectiveness of CA-125 versus TVUS as the initial investigation in the primary care setting. In addition, a diagnostic prediction model combining age and CA-125 level was shown to perform well for the detection of ovarian cancer in symptomatic UK patients (Funston, Hamilton, *et al.*, 2020). This highlights an opportunity for a risk-based triage system using CA-125 to improve early diagnostic rates.

#### Biomarkers for ovarian cancer

The use of CA-125 as an initial investigation in isolation has limitations. Current NICE thresholds for an abnormal serum CA-125 level are those above 35 IU/ml (National Institute for Health and Care Excellence, 2011). However, a recent cohort study showed that out of 456 women diagnosed with ovarian cancer, 23% had a normal initial CA-125, below the threshold for referral. More worryingly, the median time from investigation to diagnosis was significantly higher in those with normal CA-125 levels (64 days) compared to those with abnormal results (35 days) (Funston *et al.*, 2020). The same database revealed that the current NICE threshold for CA-125 demonstrated a sensitivity of 77.0% (95% CI 72.8, 80.8%) and a specificity of 93.8% (95% CI 93.6, 94.0) for ovarian cancer. However, CA-125 was shown to perform much better for the detection of invasive ovarian cancer, with a sensitivity of 85% overall and 87% in the ≥ 50 group (Funston *et al.*, 2020). Notably, a combined measure of CA-125 and Human Epididymis Protein 4 (HE4), a protein found to be overexpressed in ovarian tumours, appears to provide the most compelling evidence with a AUC of 0.91 to 0.96 (Dochez *et al.*, 2019). Further research is needed into effective biomarkers.

#### COVID-19

COVID-19 posed many challenges within oncology, from the suspension of cancer screening during the national UK lockdown and delayed routine diagnostic work (Maringe *et al.*, 2020). Results from a UK population-based study demonstrate in the four major cancers within the UK, avoidable pre-diagnostic delays due to the first pandemic wave are estimated to have caused 3620 cancer deaths, translating to a £103.8 million loss (Gheorghe *et al.*, 2021). For ovarian cancer specifically, data shows that the number of urgent referrals for ovarian cancer dropped by 60% (NHS England, 2020).

Medical research has also been adversely impacted by the pandemic; it is vital that these research efforts receive appropriate funding as we move into a post-pandemic recovery phase (Alam, Rampes and Ma, 2021).

### Opportunities

#### Identification of best practice from national audits

National audits collect and analyse data from local healthcare services to improve understanding of the national standard of care for a particular condition. This allows pockets of excellence to be identified, promoting similar progress throughout the country. The ovarian cancer audit feasibility pilot (OCAFP) collates routinely collected data for women diagnosed between 2016 and 2018 and found significant geographic variation in the proportion of patients diagnosed at early and late stages and overall survival rates (Public Health England, 2020).

Moving forward, national ovarian cancer audit should proactively collect comprehensive data on ethnicity. COVID-19 has brought to the forefront health inequalities, and it is vital that comprehensive demographic data including ethnicity is collected to allow for monitoring and action to be taken towards greater health equity (Wynia, Ivey and Hasnain-Wynia, 2010). Additional factors such as stigma, language barriers and cultural beliefs also contribute to variation between ethnic groups and can result in delayed presentation, affecting early diagnosis and referral rates (Martins and Hamilton, 2016).

#### Primary care intervention to facilitate GP behavioural change

Of particular interest for facilitating change in GP behaviour is an ongoing programme designed to develop and evaluate a primary care intervention to expedite the diagnosis of symptomatic cancer. The program is called WICKED, which stands for the Wales Interventions and Cancer Knowledge about Early Diagnosis (Stanciu *et al.*, 2018). The intervention developed from the program is a workshop consisting of independent sessions for non-clinical and clinical staff focused on cancer awareness and early diagnosis, respectively. This will be followed by a joint session on a practice-specific Cancer Safety Netting Plan (CSNP). A Cancer Safety Netting Champion will be appointed, who will lead on implementation of the CSNP within the practice. The intervention will be evaluated in a RCT. The ThinkCancer! Feasibility trial began recruiting in autumn 2019, and the results are eagerly awaited.

#### Risk algorithms to improve referral rates of suspected ovarian cancer

Risk algorithms are tools which model future risk of developing ovarian cancer. They offer a chance to identify high-risk patients, to target investigations, reducing diagnostic and treatment-related morbidity. There are four clinical tools in detecting ovarian cancer which were evaluated in multiple studies and showed promising diagnostic performance, with relatively homogenous performance. These include the Goff Symptom Index, modified Goff Symptom Index, the Society of Gynaecologic Oncologists consensus criteria and the QCancer Ovarian model. Out of these four risk algorithms, QCancer (Ovarian) was the only tool which was externally validated in a primary care setting (Funston *et al.*, 2020). QCancer (Ovarian) allows clinicals to calculate risk for ovarian cancer, demonstrating a 64.1% sensitivity and 90.1% specificity at the 10% risk threshold (Funston *et al.*, 2020). When validated externally, QCancer (Ovarian) was well-calibrated overall, but tended to overpredict risk, especially amongst older women (Collins and Altman, 2013).

In addition, QCancer (Female) and QCancer (Male) are other algorithms which calculate the risk of an undiagnosed cancer across multiple tumour sites at once. This helps to avoid the phenomenon of clinical reminder alert fatigue, making it more likely to be integrated and used in primary care (Backman *et al.*, 2017). QCancer (Female) is an algorithm developed based on large primary care datasets and demonstrates good discrimination with a ROC value of 0.84 for ovarian cancer (Hippisley-Cox and Coupland, 2013). A project collaboration between Macmillan and BMJ Informatica to explore the use of cancer decision support tools in primary care revealed that in 23% of patients found to have ovarian cancer, the QCancer algorithm had predicted a higher perceived ovarian cancer risk than their GPs (Moffat, Ironmonger and Green, 2014). Although there was no conclusive evidence that usage of the QCancer tool increased specialist referral for suspected cancer, important lessons were learned from a quality improvement perspective. The findings centred around: (1) ease of installation and continuing technical support, (2) comprehensive training to ensure GPs understand how the scores are calculated and (3) training and support on inclusive practise to ensure that clinical decisions tools do not compromise the GP–patient interaction (Moffat, Ironmonger and Green, 2014).

Another promising risk algorithm is the CanRisk Tool, an online ovarian cancer risk model incorporating the latest version of the BOADICEA (Breast and Ovarian Analysis of Disease Incidence and Carrier Estimation Algorithm) (Carver *et al.*, 2021). It is the first comprehensive epithelial tubo-ovarian risk prediction model that considers family history, polygenic risk scores, genetic and epidemiological risk factors (Lee *et al.*, 2021). Following a sample of 202 638 women from UK Collaborative Trial of Ovarian Cancer Screening (UKCTOCS) data, results showed moderate risk prediction in those at the top quintile of predicted risk. However, there was underprediction in the bottom quintile. More data will be needed to validate the full model to identify the reasons for underprediction and will need to include higher-risk groups that were excluded from the study (Lee *et al.*, 2021).

Risk algorithm tools which model future risk of ovarian cancer development could support clinical decision-making by identifying high-risk patients, targeting investigations in these populations to improve early diagnosis whilst reducing overtesting in lower risk groups. A cross-sectional survey of UK primary care indicated that clinical support tools for cancer are only available in one-third of UK general practices. This suggest that clinical support tools are underused within the NHS, but more data is needed to determine the effectiveness of these interventions before increasing rollout of this technology (Price *et al.*, 2019). Currently, a large RCT is seeking to evaluate the diagnostic accuracy, cost-effectiveness and acceptability to patients and clinicians of cancer risk algorithms (*ISRCTN22560297*).

#### Implementation of rapid diagnostics clinics and MDCs

Within the NHS, those presenting to their GP with specific ovarian cancer symptoms such as ascites or an abdominal mass will warrant immediate referral to a specialist. However, those who present with NSCS are referred via a 2-week referral (TWW) pathway. These NSCS include weight loss, loss of appetite, abdominal pain, distention or bloating. Patients with NSCS are more likely to have prolonged and unstructured diagnostic pathways, which may be associated with worse prognosis, survival and patient experience (Pearson *et al*., 2020). Indeed, data from the NCDA revealed that 32% of patients with NSCS were diagnosed at stage 4 cancer, compared with 21% in non-NSCS (Pearson *et al*., 2020).

To improve the detection of cancer and serious conditions in patients with NSCS, the NHS has developed RDCs across England (Dolly *et al.*, 2021). This allows patients with NSCS to have multiple tests conducted over a short time frame to speed up diagnosis, improve patient experiences and clinical outcomes. RDCs have complete clinical oversight from an Internal Medicine Consultant. Data collected between 2016 and 2019 from Guy’s Hospital in London, the largest RDC in England showed that 7% of patients referred to its RDC were diagnosed with cancer, and 36% with serious non-cancerous complex conditions (Dolly *et al.*, 2021). Moving forward, health economic studies are needed to analyse the cost-effectiveness of RDCs.

**Figure 3.**
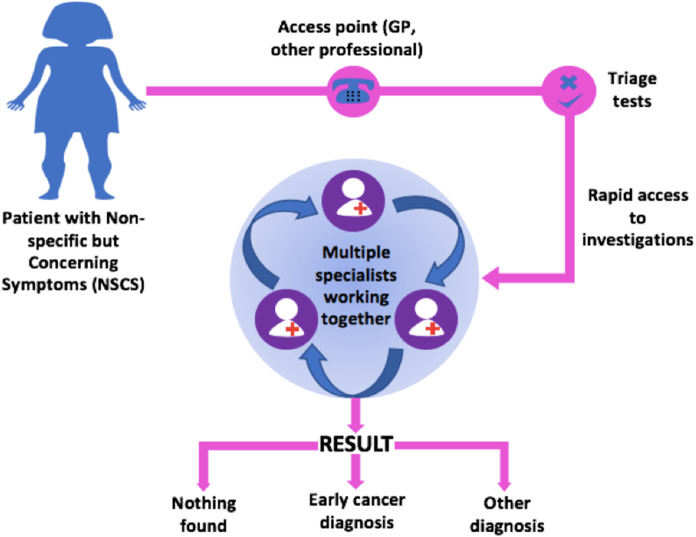
Structure of multi-disciplinary centres (MDCs).

A similar approach is taken in MDCs, where patients who present with NSCS are managed on a single diagnostic pathway, with access to diagnostic tests in rapid succession and multiple specialist consultations working together (Figure 3). MDCs could facilitate shorter primary care intervals, fewer primary care interactions before referral, earlier cancer diagnoses and fewer emergency presentations (Pearson *et al*., 2020). The Accelerate, Coordinate and Evaluate (ACE) Programme, which is a joint initiative by Cancer Research UK, Macmillan Cancer Support and NHS England is evaluating whether MDCs can result in earlier diagnosis of patients with NSCS (Chapman *et al.*, 2020). The ACE programme is structured as a series of “waves,” where each wave has its own objectives and cohort of projects (Cancer Research UK, 2020). Wave 2 was conducted between 2014 and 2019 and involved five MDC’s across England, all focussed on developing a pilot pathway for patients with NSCS. Wave 2 results appear promising with 8% of patients referred being diagnosed with different types of cancer, including ovarian cancer. Although data from the NCDA and wave 2 of the ACE programme have shown that MDCs could improve earlier diagnosis of cancer in NSCS patients, further data is needed to demonstrate the effectiveness of this new referral pathway to inform whether MDCs should be integrated into the NHS permanently.

## Conclusion

In conclusion, epidemiological research has illuminated a historically poorly understood cancer. The nature of ovarian cancer provides hurdles for GPs to recognise and refer early. Evidence confirms that UK-based GPs are less likely to refer compared to their international counterparts, and this has been correlated with lower survival rates (Rose *et al.*, 2015). Going forwards, it is of utmost importance that the potential harms of increased referral and investigations are actively considered and managed. Despite the best of intentions, increased referrals may increase health anxiety, and potentially result in over-investigation and over-treatment of women. However, these issues are much more of a concern with national screening programmes, and the consensus is that the benefits of expediting symptomatic cancer diagnosis significantly outweigh the risk of overdiagnosis. The identification of best practice from the Ovarian Cancer Audit Feasibility Pilot, the ThinkCancer! Feasibility trial, risk algorithms and MDCs are exciting opportunities to facilitate earlier diagnosis. The battle against cancer is multi-faceted, and although this review has solely addressed early diagnosis in primary care, the most successful approach will be that of a holistic one that also considers public awareness campaigns and further research into screening programmes.
