# Early diagnosis of symptomatic ovarian cancer in primary care in the United Kingdom: opportunities and challenges - ADDENDUM

**DOI:** 10.1017/S1463423625100194

**Published:** 2025-05-23

**Authors:** Sanketh Rampes, Anita Bolina, Shern-Ping Choy

The authors acknowledge resolution of disputed authorship of this article through the addition of Dr Anita Bolina to the list of authors. This addition follows an investigation conducted by the Research Governance Ethics & Integrity office at King’s College London, which concluded that Dr Bolina revised the work critically for important intellectual content. The review was undertaken with the consent of the Cambridge University Research Integrity Office.

Dr Bolina agrees to be accountable for all aspects of the work, alongside Dr Sanketh Rampes and Dr Shern-Ping Choy. Dr Bolina has no conflicts of interest or additional funding to declare.

On page 1, the authorship list reads “Sanketh Rampes and Shern-Ping Choy.” The correct author list for the article is Sanketh Rampes, Anita Bolina and Shern-Ping Choy.

On page 1, a statement read “Sanketh Rampes and Shern-Ping Choy are joint first authors”. This statement has been removed.

On page 6, the Acknowledgements section stated, “We would like to thank Dr Anita Bolina for her critical comments during manuscript preparation.” This sentence has been removed.

The article has been updated in both PDF and online versions.
